# Transgenic mouse model expressing P53^R172H^, luciferase, EGFP, and KRAS^G12D^ in a single open reading frame for live imaging of tumor

**DOI:** 10.1038/srep08053

**Published:** 2015-01-27

**Authors:** Hye-Lim Ju, Diego F. Calvisi, Hyuk Moon, Sinhwa Baek, Silvia Ribback, Frank Dombrowski, Kyung Joo Cho, Sook In Chung, Kwang-Hyub Han, Simon Weonsang Ro

**Affiliations:** 1 Liver Cirrhosis Clinical Research Center, Yonsei University College of Medicine, Seoul, Korea; 2 Brain Korea 21 Project for Medical Science College of Medicine, Yonsei University, Seoul, Korea; 3 Institute of Pathology, University Medicine Greifswald, Greifswald, Germany; 4 Department of Internal Medicine, Yonsei University College of Medicine, Seoul, Korea

## Abstract

Genetically engineered mouse cancer models allow tumors to be imaged *in vivo* via co-expression of a reporter gene with a tumor-initiating gene. However, differential transcriptional and translational regulation between the tumor-initiating gene and the reporter gene can result in inconsistency between the actual tumor size and the size indicated by the imaging assay. To overcome this limitation, we developed a transgenic mouse in which two oncogenes, encoding P53^R172H^ and KRAS^G12D^, are expressed together with two reporter genes, encoding enhanced green fluorescent protein (EGFP) and firefly luciferase, in a single open reading frame following Cre-mediated DNA excision. Systemic administration of adenovirus encoding Cre to these mice induced specific transgene expression in the liver. Repeated bioluminescence imaging of the mice revealed a continuous increase in the bioluminescent signal over time. A strong correlation was found between the bioluminescent signal and actual tumor size. Interestingly, all liver tumors induced by P53^R172H^ and KRAS^G12D^ in the model were hepatocellular adenomas. The mouse model was also used to trace cell proliferation in the epidermis via live fluorescence imaging. We anticipate that the transgenic mouse model will be useful for imaging tumor development *in vivo* and for investigating the oncogenic collaboration between P53^R172H^ and KRAS^G12D^.

Imaging of tumors *in vivo* allows the tumor to be investigated directly in its natural environment, providing invaluable information that could not be obtained otherwise. Although micro-computed tomography and micro-positron emission tomography have been used for *in vivo* imaging of tumors in small animals, high cost and technical difficulties have limited the adoption of these imaging techniques by researchers^1^. Optical imaging techniques based on fluorescence and bioluminescence signals are cost-effective and easy to use, and are therefore used widely in various fields of cancer research. For example, firefly luciferase has been applied to monitor changes in tumor sizes *in vivo* following transplantation of luciferase-expressing tumor cells into immune-deficient mice^2^. Green fluorescent protein (GFP) has also been used successfully to monitor tumor growth and metastasis *in vivo* following transplantation of GFP-expressing cancer cells^3^,^4^.

Because of the utility of reporter transgenes in imaging studies, transgenic mouse models have been developed in which a gene encoding luciferase or GFP is co-expressed with a tumor-initiating oncogene, thus allowing tumor developments to be monitored via bioluminescence or fluorescence^5^,^6^,^7^,^8^,^9^. An oncogene and a reporter gene can be co-expressed from separate promoters^5^,^7^,^8^, or a single promoter via internal ribosome entry site (IRES)-mediated expression^6^,^9^. One limitation of both approaches is that expression of an oncogene does not always accompany expression of a reporter gene, owing to, for example, differential transcriptional regulation between the separate promoters, or variation in IRES-mediated expression^10^,^11^. Thus, the reliability of the reporter in the transgenic mouse models is sometimes called into question.

Recently, the 2A sequence was used successfully to express multiple transgenes in a single open reading frame by placing it between transgenes^12^,^13^,^14^. The 2A sequence encodes a short peptide (2A peptide) of ~20 amino acids and was originally found in picornaviruses, in which multiple proteins are derived from a large polyprotein. Formation of a peptide bond at a specific site within the 2A peptide is inhibited during the translational process, resulting in the release of the preceding protein, while ribosomes continue the translation for synthesis of the next protein. Notably, stoichiometric production has been verified among proteins co-expressed from a 2A sequence-containing multi-cistronic vector, suggesting that the expression levels of a reporter gene co-expressed with a gene of interest via 2A-mediated ribosome skipping may faithfully represent the expression levels of the gene of interest^12^.

Here, we report a transgenic mouse model for cancer, in which 2A-mediated co-expression is used to express two oncogenes together with two reporter genes encoding enhanced GFP (EGFP) and firefly luciferase. Upon DNA excision by Cre, all four transgenes are expressed. We selected oncogenes encoding a constitutively active form of KRAS (KRAS^G12D^) and a gain of function mutant of P53 (P53^R172H^), to efficiently induce tumors. KRAS^G12D^ leads to constitutive activation of the Ras signaling pathway, and is associated with various types of human cancer^15^. P53^R172H^ (the murine equivalent of human P53^R175H^) is known to display novel oncogenic properties during cancer progression and promotion^16^,^17^,^18^,^19^. In this study, we also investigated the oncogenic collaboration between P53^R172H^ and KRAS^G12D^ in the liver.

## Results

### Generation of the 2PLEASE mouse (2A-connected P53^R172H^, Luciferase, EGFP, and KRAS^G12D^ expression induced by CRE)

With the goal of performing live imaging of tumor growth both microscopically and macroscopically, we employed EGFP together with firefly luciferase (hereafter termed “luciferase”) as reporters. A constitutively activated form of RAS, KRAS^G12D^ was selected as the tumor-initiating oncogene. Owing to the inefficiency of tumor induction by activated RAS alone, a gain-of-function mutant P53^R172H^ was selected as a collaborating partner for KRAS^G12D^, because of its various anticipated roles in tumor development^20^,^21^. To validate the increased tumorigenic potential of co-expressed P53^R172H^ and KRAS^G12D^, we performed a soft agar assay for anchorage-independent cell growth and a proliferation assay under low serum conditions using cell lines stably expressing both oncogenes. Co-expression of P53^R172H^ and KRAS^G12D^ rendered cells proliferative under both assay conditions (see Supplementary Fig. S1 online), suggesting that they acquired the characteristics of tumor cells. Expression of P53^R172H^ or KRAS^G12D^ alone failed to induce significant cell proliferation in either assay, justifying the use of both oncogenes to efficiently induce tumors in transgenic animals.

**Figure 1:**
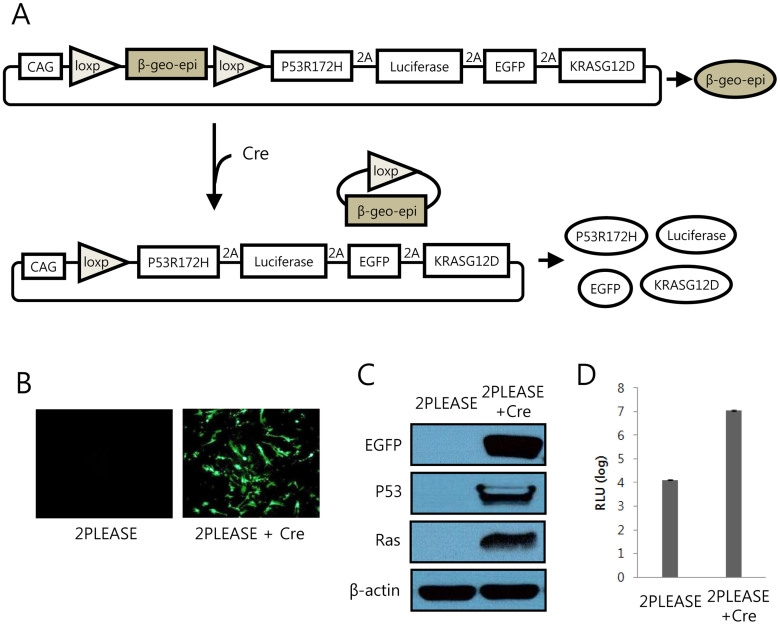
Transgene expression from the 2PLEASE plasmid upon Cre-mediated DNA excision. (A) Schematic illustration of transgene expression following Cre-mediated DNA excision. The epitope-containing β-geo (β-geo-epi) is expressed from the 2PLEASE plasmid in the absence of Cre. Following Cre-mediated DNA recombination between the two *loxP* sites, P53^R172H^, luciferase, EGFP, and KRAS^G12D^ are expressed. (B) Fluorescence imaging of cells transfected with the 2PLEASE plasmid in the absence (left) and presence (right) of Cre. (C) Western blotting shows tight regulation of transgene expression of EGFP, P53 and Ras from the 2PLEASE plasmid. The blots were performed under the same experimental condition except for blotting with different primary antibodies. These blots were shown as cropped images (D) Luminescent signals from cells transfected with the 2PLEASE plasmid increased by ~1000-fold when Cre was co-expressed, compared to cells in which Cre was not co-expressed, which displayed a background level of luminescence.

To express the two oncogenes and the two reporter genes (four transgenes in total) simultaneously in a single open reading frame, the DNA sequence encoding the *Thosea asigna* virus (TaV) 2A peptide was inserted between the transgenes, in-frame (Fig. 1A). Because expression of these oncogenes from conception would likely have a detrimental effect on animal development, we chose to control expression of the transgenes using the Cre-mediated recombination strategy. Insertion of the *lacZ*-neomycin fusion gene (β-geo), flanked by *loxP* sites, immediately prior to the four transgenes would allow β-geo to be expressed until Cre induces DNA recombination between the *loxP* sites (Fig. 1A). One concern with this Cre-mediated transgene expression strategy was that abrupt expression of the reporters EGFP and luciferase in an adult tissue following Cre-mediated DNA excision might induce an immune response, leading to a cytotoxic T-lymphocyte (CTL) response directed at cells expressing the proteins^22^,^23^. To minimize the CTL response, a DNA sequence encoding the predicted immunodominant epitope regions of EGFP and luciferase in C57BL/6 mice^24^,^25^ was inserted in-frame within the β-geo gene segment, generating the “β-geo-epi” gene, which was placed immediately prior to the four transgenes (Fig. 1A). The resulting plasmid is referred to as the “2PLEASE” plasmid.

To assess whether Cre tightly regulates the switch of gene expression in this construct, cells were transfected with the 2PLEASE plasmids in the presence or absence of Cre. As shown in Fig. 1B–1D, all transgenes (i.e., genes encoding P53^R172H^, luciferase, EGFP, and KRAS^G12D^) were expressed only in the presence of Cre. This confirms that expression of the four transgenes is tightly regulated by the Cre-*loxP* system.

β-geo protein was detected successfully in the original construct by Western blotting; however, the replacement of β-geo with β-geo-epi resulted in no detectable signals of the correct size (see Supplementary Fig. S2 online). When β-geo-epi was replaced by β-geo in the same vector, the protein was detected again (Supplementary Fig. S2 online). This suggests that the insertion of the epitope in the middle of the β-geo protein likely led to mis-folding and subsequent degradation of the chimeric protein^26^,^27^. Fusion of the epitope to the N- or C-terminal of the β-geo protein would likely minimize the mis-folding. However, we chose the degradation-prone β-geo-epi construct for the microinjection experiment because we think that degradation of the β-geo protein could reduce the possible toxicity caused by ubiquitous protein expression. Microinjection of the 2PLEASE plasmids into pronuclei of the C57BL/6 genetic background resulted in three transgenic founders. The transgenic founders were bred with R26-Cre-ER^T2^ mice^28^ in which Cre can be ubiquitously activated by treatment with tamoxifen. At 15 days post-tamoxifen administration, double-transgenic offspring of each line were assayed for bioluminescence (see Supplementary Fig. S3 online). In line 20, strong bioluminescence signals were detected from the whole body, whereas signals were not detected in mice of all other lines. Thus, mice from line 20 were used for subsequent studies and are hereafter referred to as the “2PLEASE mice”. Quantitative PCR showed that the 2PLEASE mice carry five copies of the transgene (Supplementary Fig. S3 online). The transgene was stably transmitted to the next generation (up to the sixth generation, to date).

### Induction and bioluminescence imaging of liver tumors in the 2PLEASE mice

As described above, the 2PLEASE mice were designed to express two oncogenes along with reporter genes encoding EGFP and luciferase, upon Cre-mediated DNA excision. Oncogene-induced cell proliferation is expected to generate foci within tissues where fluorescent and bioluminescent signals accumulate as a result of the expression of EGFP and luciferase. Thus, a time-dependent increase in bioluminescent signals emanating from a particular location within organ can, therefore, be an indicator of a tumor growth in this model.

**Figure 2:**
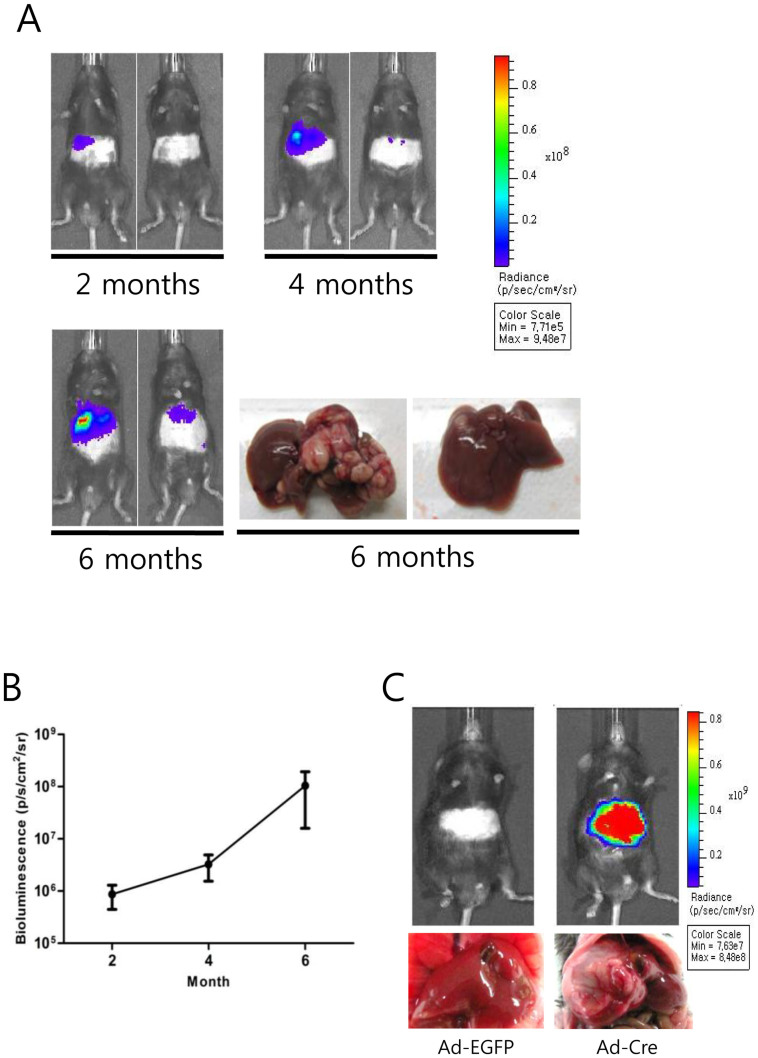
Repeated bioluminescence imaging of the 2PLEASE mice. (A) Pseudocolor images of tumor growth in the liver of 2PLEASE mice. Bioluminescence imaging was performed at 2, 4, and 6 months post-administration of adenovirus encoding Cre. Images of two representative mice are presented. Note the continuous increase in the signals from the abdominal regions of the same mice. Gross morphology of the livers harvested from the same mice (bottom right panels) confirms the correlation between the bioluminescent signals and the actual tumor sizes. (B) Average bioluminescence signals from the abdominal regions of the 2PLEASE mice (n = 23) at the indicated time-points after adenovirus injection. (C) Pseudocolor images of the abdominal regions of the 2PLEASE mice at 8 months post-treatment with control adenovirus (Ad-EGFP) and adenovirus encoding Cre (Ad-Cre). Livers harvested from the mice are presented below.

We chose the liver as the organ in which to test whether tumors can be induced by co-expression of P53^R172H^ and KRAS^G12D^, and whether repeated bioluminescence imaging of mice could trace the growth of these tumors. To induce expression of the four transgenes specifically in the liver, the 2PLEASE mice were intravenously administered a Cre-expressing adenovirus with a high tropism for hepatocytes^29^. Bioluminescence imaging, performed at 2, 4, and 6 months post-adenovirus administration, revealed increases in bioluminescent signals in the depilated abdominal areas of 12 of 23 mice (Fig. 2A,B). Notably, mice exhibiting strong bioluminescent signals showed larger tumors in the liver, compared to those with weak or moderate bioluminescent signals (see Fig. 2A and Supplementary Fig. S4 online). To test the reliability of bioluminescent signals for monitoring tumor growth further, a liver cancer model was developed via the hydrodynamic transfection method^30^. Hepatic delivery of a mixture of transposons expressing KRAS^G12D^, short hairpin RNA down-regulating p53 (shp53) and luciferase rapidly induced tumors in the liver (see Supplementary Figs. S5 and S6 online). Increases in bioluminescent signals were well correlated with actual tumor growth, further supporting the reliability of bioluminescence imaging in monitoring tumor growth (Supplementary Fig. S6 online).

The 11 mice that did not show an increase in bioluminescent signals following adenovirus administration possessed livers free from hyperplastic nodules (data not shown). No increases in bioluminescent signals or hepatic tumors were detected in mice treated with adenovirus encoding EGFP (Fig. 2C). These data indicate that transgene expression can be temporally and spatially regulated, that hepatic tumors can be induced via expression of P53^R172H^ and KRAS^G12D^, and, finally, that bioluminescence imaging can be efficiently applied to monitor tumor growth in the 2PLEASE mouse model.

### The characteristics of liver tumors induced by P53^R172H^ and KRAS^G12D^

**Figure 3:**
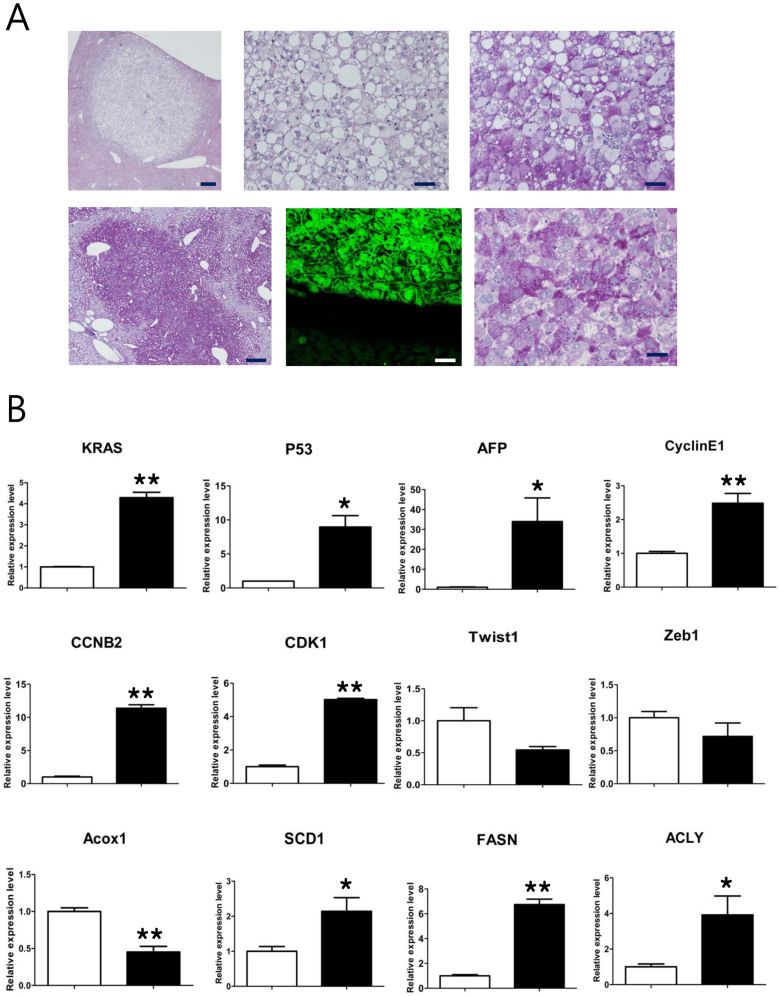
Characteristics of liver tumors harvested from 2PLEASE mice. (A) A typical focus of altered hepatocytes detected in the liver of a 2PLEASE mouse (H&E staining, upper left panel) already presenting as a mass forming hepatocellular adenoma. Higher magnification (upper middle panel) reveals enlarged hepatocytes with small lipid vacuoles. Massive glycogen storage is evident in the PAS staining (upper right panel). Another example of a glycogen-storing focus in the liver (PAS staining, lower left panel). Fluorescence imaging shows GFP expression in cells in the nodule (lower middle panel), confirming transgene expression following Cre-mediated DNA excision. Beneath the region containing green fluorescent cells are normal hepatocytes that are GFP negative. Hepatocytes in this focus are also PAS-reactive (lower right panel). Scale bars, 250 μm for left panels and 50 μm for middle and right panels. (B) Comparison of mRNA levels of selected genes. Quantitative RT-PCR was performed to compare the expression levels of the indicated genes between normal liver tissue (white bar) and liver tumors (black bar). Relative expression levels are shown. Single asterisks indicate p < 0.05 and double asterisks indicate p < 0.01.

Histological examination of liver tumors harvested from the 2PLEASE mice revealed mass-forming foci of altered hepatocytes. The lesions showed the typical morphology of glycogen-storing foci. Hepatocytes in the foci were enlarged, owing to massive glycogen storage (as indicated by a positive periodic acid-Schiff (PAS) reaction) and the presence of fat vacuoles (Fig. 3A). All tumor lesions examined were diagnosed as hepatocellular adenomas, based on published criteria^31^. Progression to carcinoma was not observed, even in tumors of ~1 cm in diameter that were harvested at 8 months post-adenovirus administration.

To investigate the molecular characteristics of the tumors, the expression levels of selected genes were analyzed by quantitative RT-PCR (qRT-PCR) (Fig. 3B). Overexpression of P53 and KRAS was confirmed in tumors from 2PLEASE mice, compared to normal livers, presumably owing to the transgenic expression of P53^R172H^ and KRAS^G12D^. The tumors also overexpressed cell cycle-related genes, such as CyclinE1, CCNB2 and CDK1, indicative of elevated cellular proliferation. Consistent with the increase in fat vacuoles in the tumors, up-regulation of genes related to lipogenesis (SCD1, FASN, and ACLY) was detected. Down-regulation of Acox1, a gene involved in lipid catabolism, was observed in the tumors. No significant differences were found between tumors and normal tissues in the mRNA levels of genes related to the epithelial-mesenchymal transition (Twsit1 and Zeb1). Interestingly, the expression level of α-fetoprotein (AFP), a putative hepatocellular carcinoma (HCC) marker, was significantly higher in tumors compared to normal liver tissue (p < 0.05). This is not consistent with the histopathological results, which found no signs of HCC in all lesions investigated. Because AFP can be up-regulated by various pathological conditions^32^,^33^, its reliability as a HCC marker should be carefully considered.

### An alternative tumor model expressing P53^R172H^ and KRAS^G12D^ shows liver tumor incidences and histological features similar to those of the 2PLEASE model

**Figure 4:**
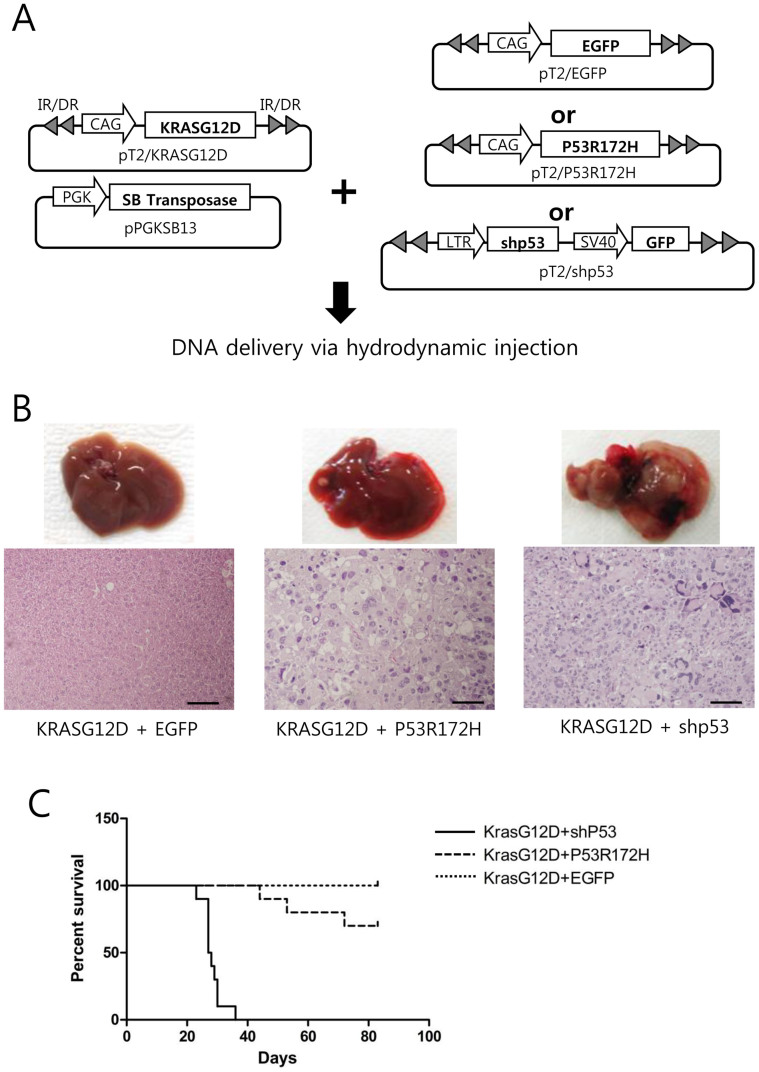
Generation of liver-specific transgenic models via the hydrodynamic transfection method. (A) Schematic illustration of the experimental procedure to generate transgenic livers expressing KRAS^G12D^ plus EGFP (control), KRAS^G12D^ plus P53^R172H^, and KRAS^G12D^ plus shp53. The indicated plasmids were mixed and hydrodynamically delivered to the liver. (B) Gross morphology and H&E staining of a representative liver from each group. While the control group shows no altered hepatocytic foci (upper left panel), single, small nodules were found in the livers of 50% of the KRAS^G12D^ plus P53^R172H^ mice at 3 months post-hydrodynamic injection (upper middle panel). KRAS^G12D^ plus shp53 mice at 1 month post hydrodynamic injection had numerous hepatocellular tumors (upper right panel) characterized by high pleomorphism and dedifferentiation of malignant hepatocytes (lower right panel). Scale bars, 100 μm. (C) Kaplan–Meier survival curves of mice expressing shp53, P53^R172H^ and EGFP in addition to KRAS^G12D^. Differences in survival were highly significant between the KRAS^G12D^ plus shp53 and KRAS^G12D^ plus P53^R172H^ groups (p < 0.0001).

Expression of P53^R172H^ and KRAS^G12D^ in the livers of the 2PLEASE mice did not efficiently induce tumors; an incidence of ~50% was observed by 6 months post-adenovirus administration. Furthermore, the tumors that did arise failed to progress to carcinoma. The adenoviral vector used for Cre expression may have induced an immune response to cells infected with the virus. In addition, although they are fewer than 20 amino acids long, truncated 2A peptides attached to P53^R172H^ and KRAS^G12D^ may have modified the function of the onco-proteins. To rule out the possibility that the adenovirus or 2A peptides influenced tumor development, we utilized an alternative transgenic mouse model simultaneously expressing P53^R172H^ and KRAS^G12D^. The alternative model was developed via a hydrodynamics-based transfection method, coupled with the *Sleeping Beauty* transposon system, which we have successfully used previously to generate various transgenic models for liver cancer^30^,^34^. Transposons encoding P53^R172H^ and KRAS^G12D^ were mixed with plasmids expressing the *Sleeping Beauty* transposase and then hydrodynamically delivered to the liver (Fig. 4A). Livers harvested at 3 months post-hydrodynamic injection (PHI) contained hyperplastic nodules in 50% of mice (Table 1). Tumors induced by P53^R172H^ and KRAS^G12D^ in this model showed few signs of malignancy; similar to tumors found in the 2PLEASE model (Fig. 4B middle panels). Thus, it is not likely that the adenovirus and the 2A peptide used in the 2PLEASE mouse affected tumor development in the liver.

In contrast, highly malignant liver tumors developed in mice expressing KRAS^G12D^ and short hairpin RNA down-regulating p53 (shp53) by 1 month PHI (Fig. 4B right panels). These tumors were solid and diagnosed as malignant hepatocellular carcinomas. Tumor cells were extremely pleomorphic with highly atypical nuclei. The tumors contained cells of highly undifferentiated morphology and many giant tumor cells. Kaplan–Meier survival analysis also revealed the existence of a highly significant difference in survival between mice expressing KRAS^G12D^ plus shp53 and those expressing KRAS^G12D^ plus P53^R172H^ (Fig. 4C, p < 0.0001). No tumors were found in livers expressing KRAS^G12D^ and EGFP (Fig. 4B left panels), implying that KRAS^G12D^ alone cannot induce liver tumors. Furthermore, no pre-neoplastic or neoplastic lesions were found in livers expressing P53^R172H^ or shp53 alone (data not shown).

**Figure 5:**
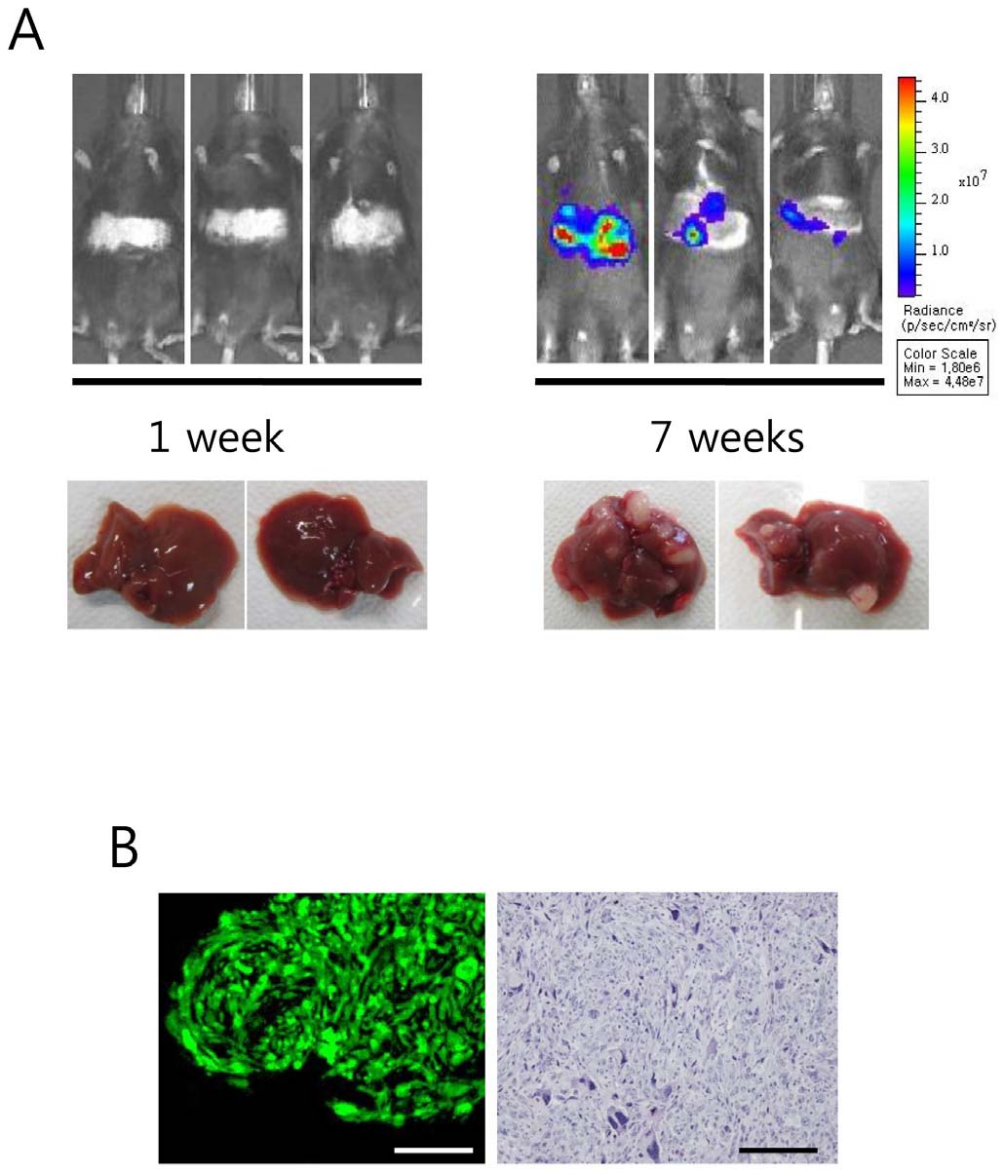
Liver tumor development was enhanced in the 2PLEASE model by the suppression of wild-type p53. (A) Bioluminescence imaging of 2PLEASE mice was performed at 1 and 7 weeks post-hydrodynamic delivery of transposons co-expressing shp53 and Cre. Gross morphologies of livers harvested at the time points are shown below. (B) Green fluorescence imaging of a representative tumor harvested at 7 weeks post-hydrodynamic injection (left panel) and H&E staining of the tumor (right panel). Scale bars, 200 μm.

To enhance liver tumor development in the 2PLEASE model, suppression of wild-type p53 was attempted via hydrodynamic delivery of transposons expressing shp53. To express P53^R172H^ and KRAS^G12D^ (via Cre-mediated transgenic switching) together with shp53 in the same cells, a chimeric transposon vector was employed that expresses both shp53 and Cre (see Supplementary Fig. S5 online). Tumor development was increased dramatically in the liver as multiple large tumor nodules were observed at 7 weeks post-hydrodynamic transfection (Fig. 5A). Bioluminescence imaging revealed increases in signal intensity from the abdominal area over the 7-week period and corresponded well to tumor development (Fig. 5A). Strong green fluorescence was observed from tumors *ex vivo*, confirming transgenic expression in the 2PLEASE mice following Cre-mediated DNA excisions (Fig. 5B left panel). Histopathologic examination revealed that all tumors consisted of hepatocellular carcinomas and the tumor cells were highly undifferentiated with atypical nuclei (Fig. 5B right panel). Thus, the down-regulation of wild-type p53 together with the expression of P53^R172H^ and KRAS^G12D^ dramatically accelerated tumor growth and promoted hepatocellular carcinomas.

### Induction of transgene expression in skin cells of 2PLEASE mice

**Figure 6:**
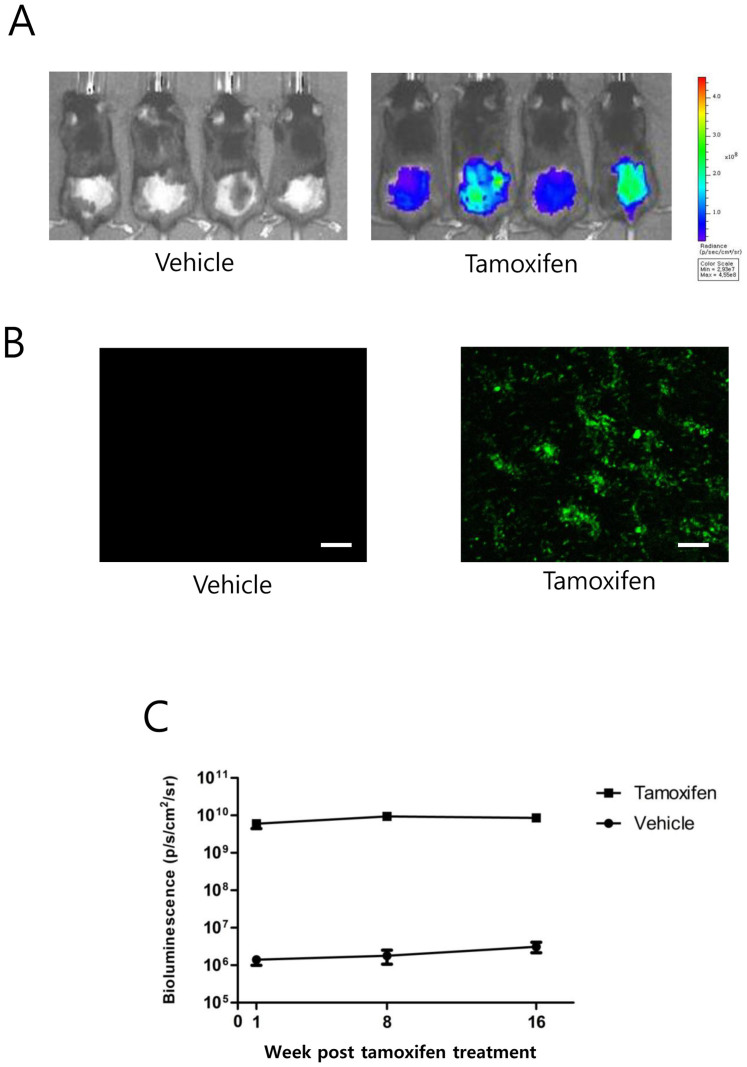
Robust transgene expression in the skin following Cre-mediated DNA excision. (A) Bioluminescence imaging of the dorsal skin of 2PLEASE; R26-Cre-ER^T2^ double-transgenic mice at 1 week post-treatment with vehicle (left panel) and tamoxifen (right panel). (B) Basal cells in the dorsal skin of these mice were imaged via *in vivo* green fluorescence imaging. Numerous skin cells in the basal layer expressed EGFP following tamoxifen treatment, while no detectable signals were found in the layer of vehicle-treated mice (Tile-scanned images, scale bars, 200 μm). (C) Average bioluminescent signals from the dorsal skin of mice at the indicated time points after topical treatment with tamoxifen or vehicle. Note that no increases in bioluminescence signals were observed from the tamoxifen-treated mice over several months.

To investigate whether expression of P53^R172H^ and KRAS^G12D^ is capable of inducing skin cancer and whether tumor growth in skin can be monitored via *in vivo* imaging of the co-expressed reporters, 2PLEASE mice were crossed with R26-Cre-ER^T2^ mice and the double transgenic offspring (2PLEASE; R26-Cre-ER^T2^) were treated topically with tamoxifen on the dorsal skin following depilation. Bioluminescence imaging performed at one week post-treatment revealed strong signals on the depilated skin of the tamoxifen-treated mice, while background signals were detected from vehicle-treated mice (Fig. 6A). Furthermore, *in vivo* fluorescence imaging showed that epidermal cells on the basal layer of tamoxifen-treated mice exhibited green fluorescence (Fig. 6B). Thus, bioluminescence and *in vivo* fluorescence imaging confirmed robust expression of the transgenes in the skin following treatment with tamoxifen. However, no increases in bioluminescent signals were observed over several months in the tamoxifen-treated skin, strongly suggesting that the tumor did not develop in the skin (Fig. 6C). Consistent with the bioluminescence imaging data, no tumors were detected on the skin of 11 double-transgenic mice until 8 months post-tamoxifen treatment (data not shown). Interestingly, one mouse, excluded from the *in vivo* imaging experiment due to a severe injury to the depilated skin, developed skin tumors at about 2 months post-tamoxifen treatment (see Supplementary Fig. S7 online). Live imaging showed strong bioluminescence and green fluorescence signals from the tumor (Supplementary Fig. S7 online). Histo-pathologic examination revealed that the tumor was a well-differentiated squamous cell carcinoma of the skin. These data suggest that co-expression of KRAS^G12D^ and P53^R172H^ is not sufficient to induce skin tumors, which might require an additional tumor-promoting condition, such as inflammation, for efficient development of skin cancer^35^,^36^.

### Imaging of clonal development of epidermal cells following transgene expression

Previously, we developed the Stop-EGFP mouse, in which EGFP is randomly expressed in skin cells, and successfully used the mouse to trace clonal growth of epidermal stem cells via *in vivo* fluorescence imaging^37^,^38^. We therefore attempted to trace a clonal lineage of skin cells expressing P53^R172H^ and KRAS^G12D^ by means of fluorescence imaging of co-expressed EGFP. For this purpose, a low dose of tamoxifen was applied topically to depilated skin of double-transgenic (2PLEASE; R26-Cre-ER^T2^) mice to reduce the frequency by which Cre induced DNA recombination (i.e., transgenic switching), thus guaranteeing the clonality of observed cell lineages.

**Figure 7:**
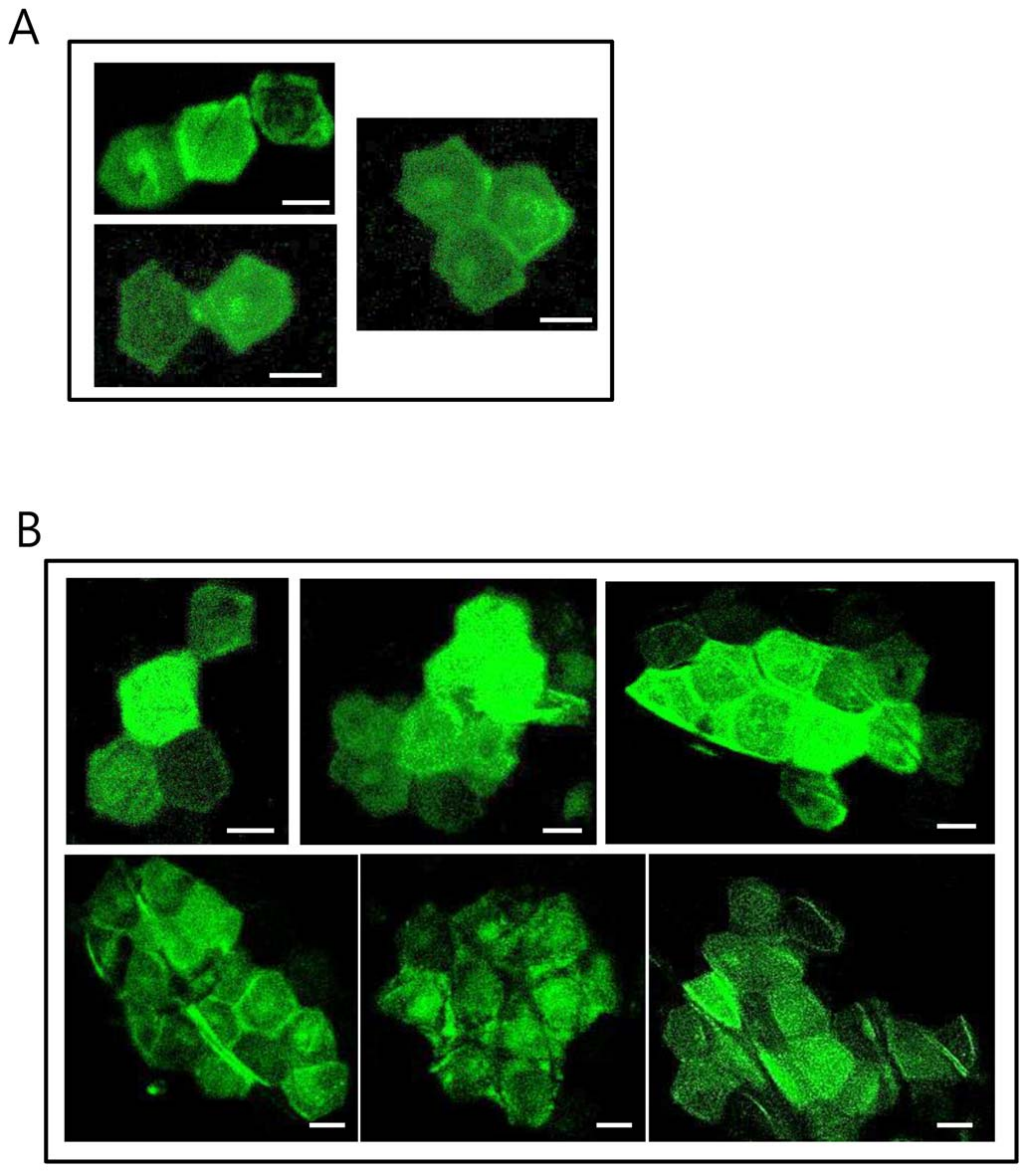
In vivo imaging of green fluorescent cells in the dorsal skin. (A) Green fluorescent patches containing corneocytes were observed via live fluorescence imaging of the dorsal skin of double-transgenic mice (2PLEASE; R26-Cre-ER^T2^) one month after topical treatment with a low dose of tamoxifen. The hexagonal shape is characteristic of the morphology of a cornified cell (corneocyte) in the outermost layer of the epidermis. (B) At 4 months post-treatment, in vivo imaging of the same area of skin occasionally revealed an increase in the size of the fluorescent region, which was found to contain as many as 20 green fluorescent corneocytes. Scale bars, 20 μm.

One month after the low-dose tamoxifen application, *in vivo* fluorescence imaging revealed several green fluorescent patches, each containing between one and four adjacent corneocytes, within a skin area of 1 × 1 cm (Fig. 7A). No green fluorescent cells were observed in the skin of vehicle-treated double transgenic offspring, or 2PLEASE mice treated with tamoxifen (data not shown).

At 4 months post-tamoxifen administration, repeated imaging of the same skin area revealed green fluorescent patches that were generally similar in size to those seen at the earlier time-point (i.e., between one and four adjacent corneocytes on the skin surface). However, larger patches containing as many as 20 corneocytes (Fig. 7B) were occasionally observed, indicative of proliferation and clonal expansion of epidermal cells expressing KRAS^G12D^ and P53^R172H^ since the initial imaging phase. The data suggest that P53^R172H^ and KRAS^G12D^ can promote cellular proliferation in the skin although the oncogenes are not strong enough to induce skin tumors.

## Discussion

In this study, we developed a transgenic tumor model in which two onco-proteins (P53^R172H^ and KRAS^G12D^) and two reporters (firefly luciferase and EGFP) are expressed from a single open reading frame upon Cre-mediated DNA excision. Liver tumors were induced by the expression of P53^R172H^ and KRAS^G12D^ in 2PLEASE mice following Cre-mediated DNA excision and subsequently imaged *in vivo* via the co-expressed reporters. The bioluminescent signals were well correlated with actual tumor sizes, demonstrating the utility of this novel mouse model for live imaging of tumors.

Tumors in the liver expressing P53^R172H^ and KRAS^G12D^ failed to progress to carcinoma, whereas P53 inactivation (via short hairpin RNA) in combination with KRAS^G12D^ expression induced highly malignant carcinoma. The reason for the apparent inefficiency of P53^R172H^ to cooperate with Kras^G12D^ to induce hepatic carcinoma is unknown from our study. P53^R172H^ is known to function as a gain-of-function mutant, rather than simply inactivating wild-type P53 as a dominant negative mutant^18^,^19^. Lines of evidence have shown that the spectra and characteristics of tumors induced by P53^R172H^ are quite different from those of tumors induced by inactivation of wild-type *p53*^16^,^17^. Of note, the tumorigenic function of P53^R172H^ seems highly dependent on the presence of wild-type P53. P53^R172H^ knock-in models feature inefficient tumor induction in mice heterozygous for P53^R172H^ (*P53^R172H/+^*, thus maintaining one allele of the wild-type *p53*), compared to homozygous mice (*P53^R172H/R172H^*, thus containing no wild-type *p53* alleles)^16^,^17^. Using a knock-in mouse model heterozygous for P53^R172H^ (*P53^R172H/+^*) Tuveson et al. showed that all of the pancreatic tumors that developed in the mice displayed *p53* loss of heterozygosity (LOH)^20^, resulting in the deletion of the wild-type allele of *p53*. Thus, the presence of the wild-type allele of *p53* would likely inhibit the oncogenic properties of P53^R172H^.

To suppress wild-type *p53* with the transgene expression simultaneously, 2PLEASE mice were hydrodynamically injected with transposon vectors co-expressing shp53 and Cre. Of note, liver tumors developed rapidly within 2 months with concomitant increases in bioluminescent signals and all progressed to malignant hepatocellular carcinomas. To study the molecular pathways promoting the development of hepatocellular carcinoma and/or accelerate tumor formation, similar composite transposon vectors can be developed that co-express another oncogene (such as cMyc or an activated β-catenin) together with Cre^39^,^40^. Hydrodynamic delivery of such transposons would enhance tumor growth in the liver of 2PLEASE mice, and can be monitored via bioluminescence imaging. Further, it could be investigated whether additional oncogenes can promote the transition from hepatocellular adenoma (induced by P53^R172H^ plus KRAS^G12D^) to carcinoma.

Although robust expression of transgenes was confirmed via *in vivo* fluorescence imaging and bioluminescence imaging in the skin (Figure 6 A, B), skin tumors did not develop in double-transgenic (2PLEASE; R26-Cre-ER^T2^) mice following topical application of tamoxifen. Interestingly, squamous cell carcinoma of the skin was found in one mouse that had a severe wound on tamoxifen-treated skin, which was accidentally caused by another male housed in the same cage. Our highly speculative idea is that injury-mediated inflammation may have promoted skin cancer in this mouse^35^,^36^. It would be interesting to see how treatment with an agent inducing skin inflammation, such as 12-*O*-tetradecanoylphorbol-13-acetate (TPA), affects the development of skin cancer in the 2PLEASE model. Using a low-dose scheme of tamoxifen treatment, we induced transgene switching in skin cells at a low frequency in 2PLEASE; R26-Cre-ER^T2^ mice. Clonal development of skin cells due to increased cellular proliferation induced by P53^R172H^ plus KRAS^G12D^ was observed via *in vivo* fluorescence imaging. It would also be interesting to investigate how these cells behave following treatment with TPA via repeated *in vivo* fluorescence imaging over time.

One potential problem with 2PLEASE mice is that they carry multiple copies of the transgenes. Because of the presence of multiple *loxP* sites, DNA recombination by Cre might result in unexpected breakdown of the transgene copies in the genome of 2PLEASE mice. The knock-in strategy will resolve this issue and is expected to refine the current 2PLEASE model^41^,^42^.

## Methods

### Plasmids

Plasmids harboring cDNA encoding firefly luciferase and EGFP were kind gifts from Dr. Rabinovich^43^ and Dr. Okabe^44^, respectively. The plasmid Z/EG, a generous gift from Dr. Lobe^45^, was modified to construct the plasmid, 2PLEASE. Construction of 2PLEASE was performed in two steps. First, to minimize the cytotoxic T-lymphocyte (CTL) response to the reporter proteins, DNA sequences encoding predicted immunodominant CTL epitopes of EGFP and firefly luciferase in C57BL/6 mice (DTLVNRIEL and LMYRFEEEL, respectively) were inserted, in-frame, into the β-geo cDNA, after digesting Z/EG with the *Msc*I restriction enzyme^22^,^23^. The resulting plasmid is referred to as Zepi/EG. Next, the cDNAs encoding the four transgenes were substituted for EGFP cDNA in the Zepi/EG. Prior to the substitution, to express the four transgenes in a single open reading frame, the termination codons located in the cDNAs encoding P53^R172H^, luciferase, and EGFP were removed, and then the DNA sequence encoding the *Thosea asigna* virus (TaV) 2A peptide with a GSG linker at the N-terminus (i.e., GSGEGRGSLLTCGDVEENPGP) was inserted, in-frame, between the cDNAs^13^.

### Transfection, Western blotting, and in vitro reporter assays

NIH3T3 cells (CRL-1658; ATCC, Manassas, USA) were transiently transfected with 1 μg of 2PLEASE plasmid and 1 μg of either pBS185CMV-Cre (ADDGENE #11916) or an empty vector (a negative control), using FuGENE HD Transfection Reagent (Promega, Madison, USA), according to the manufacturer's instructions. Cells were harvested at 2 days post-transfection and lysed in 1 × RIPA buffer (#9806; Cell Signaling). Western blotting experiments were performed using standard methods. Anti-p53 (#2524), anti-GFP (#2555) and anti-LacZ (#2372) primary antibodies were purchased from Cell Signaling Technology, and anti-Kras (sc-30) and anti-β-actin (sc-47778) were purchased from Santa Cruz Biotechnology. Horseradish peroxidase (HRP)-conjugated anti-mouse IgG (sc-2005, Santa Cruz) and anti-rabbit IgG (A0545, Sigma) were used as secondary antibodies. Fluorescence imaging of transfected cells was performed at 2 days post-transfection, using an inverted microscope (IX71, Olympus, Japan). Luciferase activity was measured using the Dual-Luciferase Reporter Assay System (Promega), according to the manufacturer's instructions.

### Generation of stable cell lines and cell growth assays

Stable cell lines were generated by transfection of NIH3T3 cells with plasmids encoding the respective oncogenes, and subsequent selection of transfected cells using a G418-containing medium. A soft agar assay for anchorage-independent cell growth and a proliferation assay under a low serum condition were performed following standard protocols^46^. For the low serum condition, Dulbecco's modified Eagle's medium (DMEM) containing 1% bovine calf serum was used.

### Animal experiments

All experiments using live mice were performed in strict accordance with the Guidelines and Regulations for the Care and Use of Laboratory Animals in AAALAC-accredited facilities, and were approved by the Animal Policy and Welfare Committee of the Yonsei University College of Medicine (Permit number: 10-026).

### Transgenic mice

The 2PLEASE constructs were digested with *Sfi*I and *Sca*I, and a DNA fragment of 12.6 kb was purified and then microinjected into fertilized one-cell pronuclei (C57BL/6N x C57BL/6N), using standard techniques. The genotypes of mice were determined by genomic PCR using primers 5′-CTCCTGACTACTCCCAGTCATAGC-3′ and 5′- GGCGGGCCATTTACCGTAAGTTAT-3′. The R26-Cre-ER^T2^ mouse was a generous gift from Dr. T. Jacks^28^.

### Adenoviral administration and bioluminescence imaging

Adenoviruses encoding Cre (Ad5CMVCre) and EGFP (Ad5CMVEGFP) were purchased from the Gene Transfer Vector Core at the University of Iowa^47^. Each mouse was injected with 1.6 × 10^8^ pfu of adenovirus through the lateral tail vein. Bioluminescence imaging was performed as described previously^34^. Briefly, the abdominal area of skin was depilated using depilatory cream 1 day before imaging. On the day of imaging, mice were intraperitoneally injected with D-luciferin (150 mg/kg) and bioluminescence imaging was performed using the IVIS Imaging System (Caliper Life Sciences, Alameda, CA, USA).

### Histopathological analysis and fluorescence imaging of tumor tissues

After euthanizing mice, their livers were removed and rinsed in phosphate-buffered saline (PBS). Samples collected from the livers were fixed overnight in freshly prepared neutral-buffered formalin. Fixed tissue samples were embedded in paraffin. Sections (5 μm) were placed on slides and stained with hematoxylin and eosin (H&E) and PAS. Liver lesions were assessed by certified pathologists in accordance with the criteria established by Frith et al^31^.

For fluorescence imaging of tumor cells in the liver, tumor-bearing mice were perfused with 10 mL of saline followed by 10 mL of 4% paraformaldehyde after anesthesia. After cutting with scissors, one part of the tumor was transferred to neutral-buffered formalin and fixed overnight followed by paraffin embedding (for H&E and PAS), and the remaining part of tumor tissue was stored in 4% paraformaldehyde at 4°C with gentle agitation for 11 hrs. The fixed tissue was stored in PBS with 1 mM MgCl_2_ at 4°C overnight and then sectioned into 100-μm-thick slices using a vibratome (VT1000S, Leica). Mounting and fluorescence imaging were performed as described previously^48^. Images were acquired using a confocal laser scanning microscope (LSM710, Zen 2009 software, Carl Zeiss) mounted on an inverted microscope (Axio Observer.Z1, Carl Zeiss). EGFP was excited with a 488-nm laser line and a band-pass filter (505–530 nm wavelength) was used to detect emission from EGFP.

### Quantitative RT-PCR

RNA was isolated from mouse liver tissue using TRIzol (Invitrogen, Carlsbad, USA), according to the manufacturer's instructions. Following treatment with 1 U/μL DNase I (Invitrogen, Carlsbad, CA, USA), 1 μg of RNA was used for reverse-transcription (Superscript III RT, Invitrogen, Carlsbad, USA), according to the manufacturer's instructions. Real-time PCR was carried out on an Applied Biosystems 7500 thermocycler using the following conditions: 95°C for 10 min, followed by 40 cycles at 95°C for 15 s and 60°C for 1 min, followed by a hold at 4°C. The sequences of the primers used for qRT-PCR are listed in Supplementary Table S1 online.

### Hydrodynamic injection

The plasmids pT2/EGFP, pT2/shp53, and pPGK-SB13 were described previously^34^. Transposon plasmids pT2/KRAS^G12D^ and pT2/P53^R172H^ were generated by replacing the EGFP cDNA in pT2/EGFP with cDNA encoding KRAS^G12D^ and pT2/P53^R172H^, respectively.

Hydrodynamic injection was performed as described previously^34^.

### Topical application of tamoxifen and *in vivo* fluorescence imaging of the skin

The hair in a 2.5 × 2.5-cm area of the lower part of the dorsal skin of the 2PLEASE mice was clipped using electric clippers (Oster, USA). A depilatory agent was applied to the clipped area for 1 min. A 100-μL aliquot of tamoxifen in ethanol (50 mg/mL) was topically applied to the depilated area for three consecutive days. For low-dose treatment, a 100-μL aliquot of tamoxifen in ethanol (10 mg/mL) was topically applied to the depilated area once. Detailed procedures for *in vivo* fluorescence imaging of EGFP-expressing cells in the skin are described elsewhere^37^,^38^. Basal cells in a large area of skin were imaged by the “Tile scan” function using the motorized scanning stage of a confocal laser scanning microscope (LSM 710, Zen 2009 software, Carl Zeiss) with a 20X lens (NA 0.8, Plan-Apochromat).

### Statistical analysis

The quantitative RT-PCR data and bioluminescence signals were expressed as the mean ± SD with sample sizes n = 3 or larger. Statistical analyses of these data were conducted via an unpaired parametric Student's t test. Kaplan–Meier survival data were evaluated using a log-rank test. A *p* value of less than 0.05 was considered statistically significant.

## Author Contributions

H.J. carried out experiments, analysed and interpreted data; D.C., S.R. and F.D. provided histopathological analysis of tissue samples; H.M., S.B., K.C. and S.C. carried out experiments; K.H. analysed and interpreted data; S.R. conceived this study, analysed and interpreted data and wrote the manuscript. All authors approved the submitted manuscript.

## Supplementary Material

Supplementary Information Supplementary Information

## Figures and Tables

**Table 1:** Tumor incidence in the liver

Genes	KRAS^G12D^ + EGFP	KRAS^G12D^ + P53^R172H^	KRAS^G12D^ + shp53
Tumor incidence*(%)	0/10 (0%)	10/20 (50%)	10/10 (100%)

*Livers were harvested at 3 months PHI for the KRAS^G12D^ + EGFP and Kras^G12D^ + P53^R172H^ groups. For the KRAS^G12D^ + shp53 group, livers were harvested at 1 month PHI because signs of illness were present.
