# Supplementary material for: Transgenic mouse model expressing P53R172H, luciferase, EGFP, and KRASG12D in a single open reading frame for live imaging of tumor
Source: Sci Rep. 2015 Jan 27;5:8053. doi: 10.1038/srep08053 (PMC4306974; doi:10.1038/srep08053)
Supplement: Supplementary Information [file srep08053-s1.pdf]

Supplementary Information

(1 Table and 8 Figures)

"Transgenic mouse model expressing $P53^{R172H}$, luciferase, EGFP, and $KRAS^{G12D}$ in a single open reading frame for live imaging of tumor"

Hye-Lim Ju[1,2], Diego F. Calvisi[3], Hyuk Moon[1,2], Sinhwa Baek[1,2], Silvia Ribback[3], Frank Dombrowski[3], Kyung Joo Cho[1], Sook In Chung[1,2], Kwang-Hyub Han[1,4], and Simon Weonsang Ro[1,4,*]

[1]Liver Cirrhosis Clinical Research Center, Yonsei University College of Medicine, Seoul, Korea

[2]Brain Korea 21 Project for Medical Science College of Medicine, Yonsei University, Seoul, Korea

[3]Institute of Pathology, University Medicine Greifswald, Greifswald, Germany.

[4]Department of Internal Medicine, Yonsei University College of Medicine, Seoul, Korea

Supplementary Table S1. Primers used for a quantitative PCR

Genes	Forward Primer Sequence (5’→3’)	Reverse Primer Sequence (5’→3’)
KRAS	TGA AGA TGT GCC TAT GGT CCT GGT	TGC TAA CTC CTG AGC CTG TTT CGT-
P53	GCT TTG AGG TTC GTG TTT GTG CCT	GGC AGT TCA GGG CAA AGG ACT TC
CDK1	ACA CCT TTC CCA AGT GGA AGC	GCC ATT TTG CCA GAG ATT CG
CyclinE1	AAG CGA GGA TAG CAG TCA GC	GGA TGA AAG AGC AGG GGT CC
CCNB2	TGT CAA CAA GCA GCC GAA AC	TCA GAG AAA GCT TGG CAG AGG
Acox1	CTC ACT CGA AGC CAG CGT TA	CGG TGC ACA GAG TTT TTA AAC CA
Twist1	CTG CCC TCG GAC AAG CTG AG	CTA GTG GGA CGC GGA CAT GG
Zeb1	GTT CTG CCA ACA GTT GGT TT	GCT CAA GAC TGT AGT TGA TG
FASN	CAA GTG TCC ACC AAC AAG CG	GGA GCG CAG GAT AGA CTC AC
SCD1	CAA ACA CCC GGC TGT CAA AG	TGA AGC ACA TCA GCA GGA GG
ACLY	GCT GCC ATG GTC TAC CCT TT	GAT CAG CAC GTC TAC CTC CG
AFP	AAA CCT CCA GGC AAC AAC CA	ACT CCA GCG AGT TTC CTT GG
GAPDH	GTG AAG GTC GGT GTG AAC GGA TTT	TGG CAA CAA TCT CCA CTT TGC CAC
β-geo	AGC GCC GAA ATC CCG AAT CTC TAT	ACA TCG CAG GCT TCT GCT TCA ATC
GAPDH (for copy # determination)	ATG GGA AGC TTG TCA TCA ACG GGA	AAG ACA CCA GTA GAC TCC ACG ACA

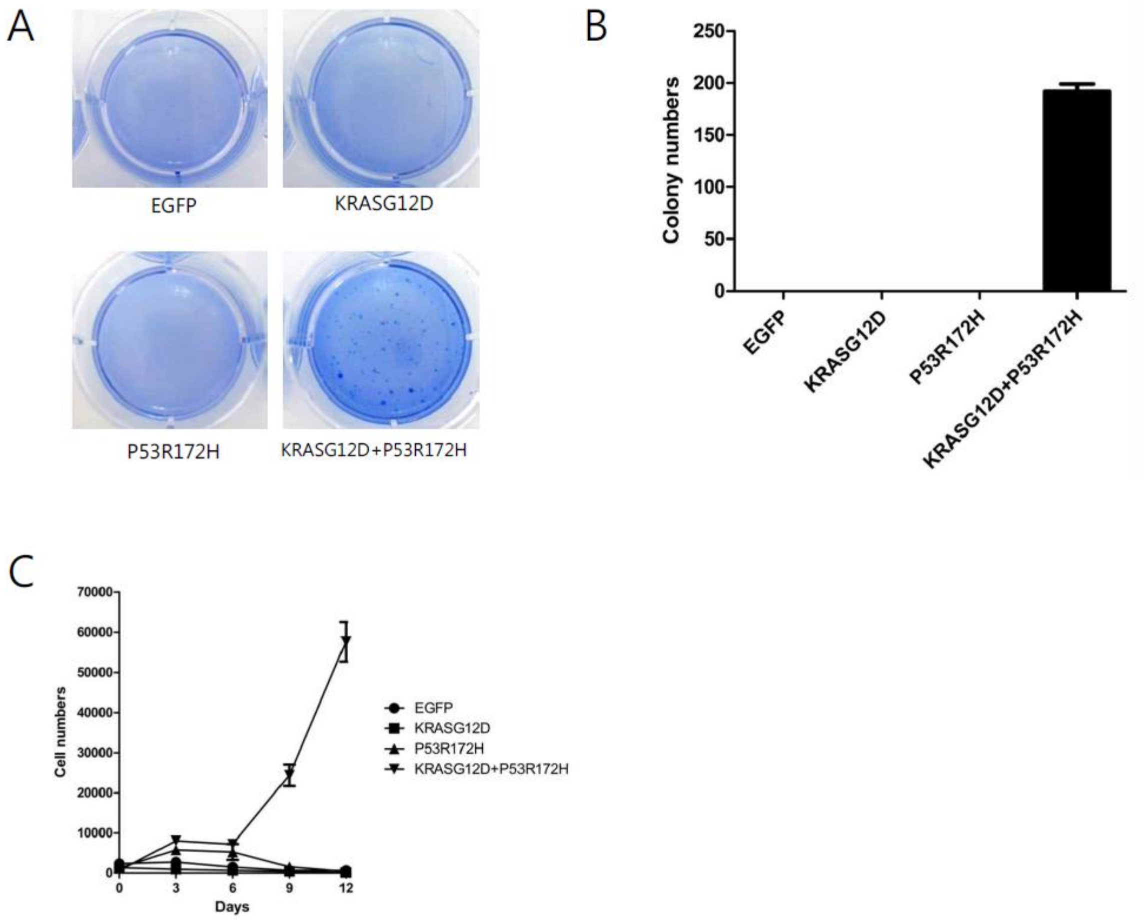

Supplementary Figure S1. Oncogenic cooperation between $P53^{R172H}$ and $KRAS^{G12D}$.

(A) A soft agar assay for anchorage-independent cell growth was performed using NIH3T3 cell lines stably expressing the indicated genes. (B) A graphic representation of the results shown in (A). Data are presented as means ± standard error of the mean. (C) A proliferation assay under a low serum condition (1% serum) showed significant growth only in the cell lines expressing $P53^{R172H}$ and $KRAS^{G12D}$. Data are presented as means ± standard error.

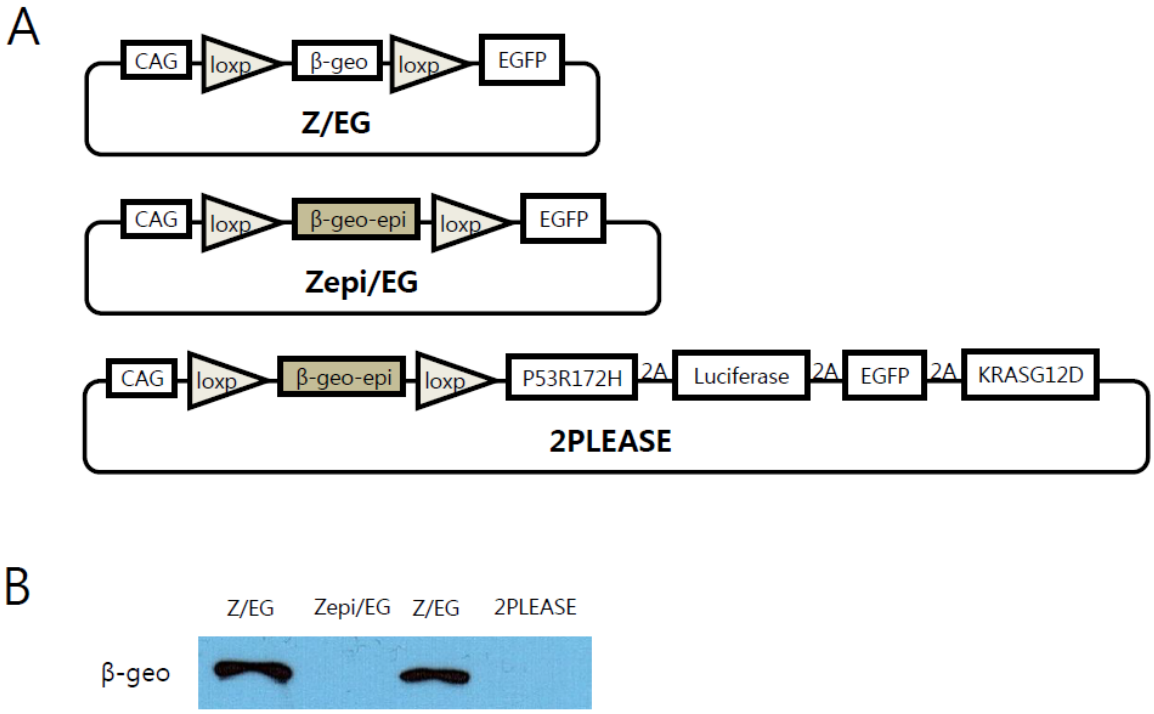

Supplementary Figure S2. Detection of β-geo by Western blotting in the absence of Cre.

(A) Plasmid maps of Z/EG, Zepi/EG and 2PLEASE. DNA sequences encoding predicted immunodominant CTL epitopes of EGFP and firefly luciferase in C57BL/6 mice were inserted in-frame into β-geo cDNA, to produce β-geo-epi. (B) Western blotting shows β-geo expression from the parental plasmid, Z/EG; however, β-geo expression was not detected after the epitope region was inserted into the β-geo protein, as seen in both Zepi/EG and 2PLEASE.

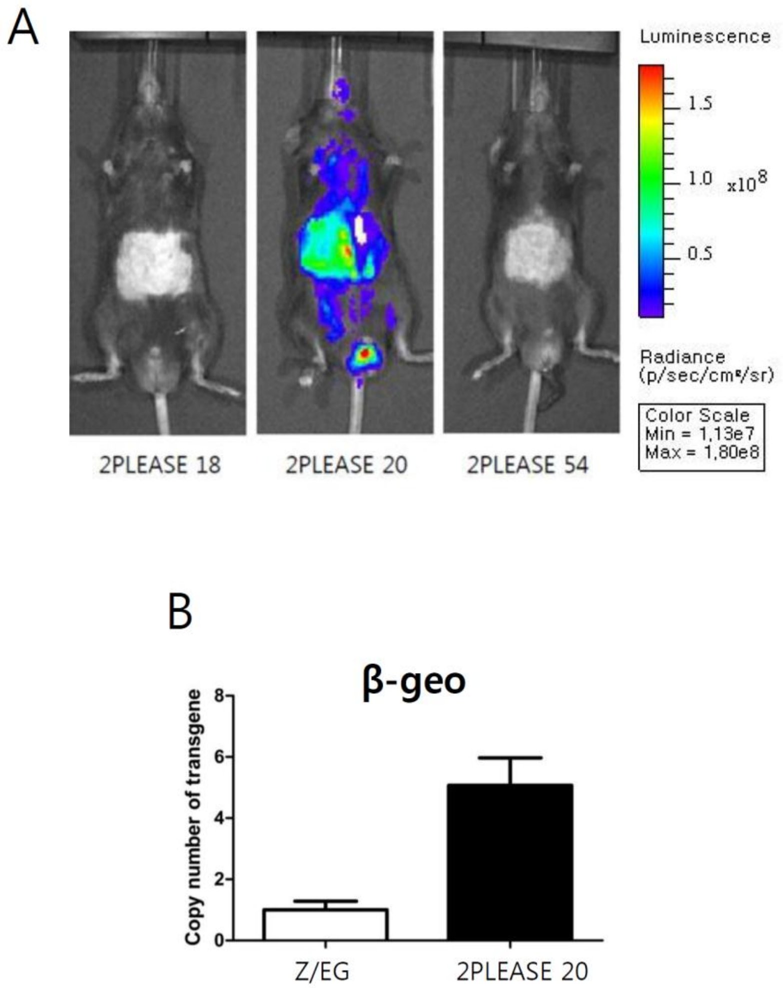

Supplementary Figure S3. Bioluminescence imaging of double-transgenic mice and determination of transgene copy number.

(A) Each line of 2PLEASE transgenic mouse was crossed with the R26-Cre-ER^{T2} mice. Double-transgenic offspring were treated with tamoxifen. Pseudocolor images of bioluminescent signals from mice from the indicated 2PLEASE lines are shown. Note the strong signals detected in the depilated abdominal area of the mouse from line 20. (B) The copy number of the transgenes was determined by the real-time PCR method via comparison of the copy number of β-geo in the 2PLEASE 20 with that in Z/EG (carrying one copy of the β-geo transgene).

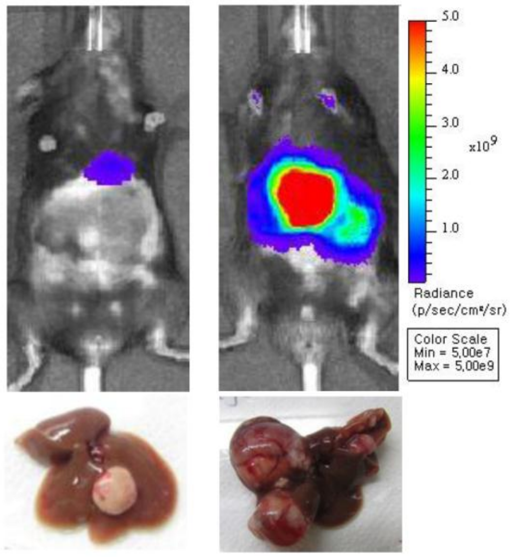

Supplementary Figure S4. Bioluminescent signals and actual tumor sizes in the livers of 2PLEASE mice.

Bioluminescence imaging was performed at 8 months post-administration of adenovirus encoding Cre. Gross morphology of the livers harvested from the mice is shown below.

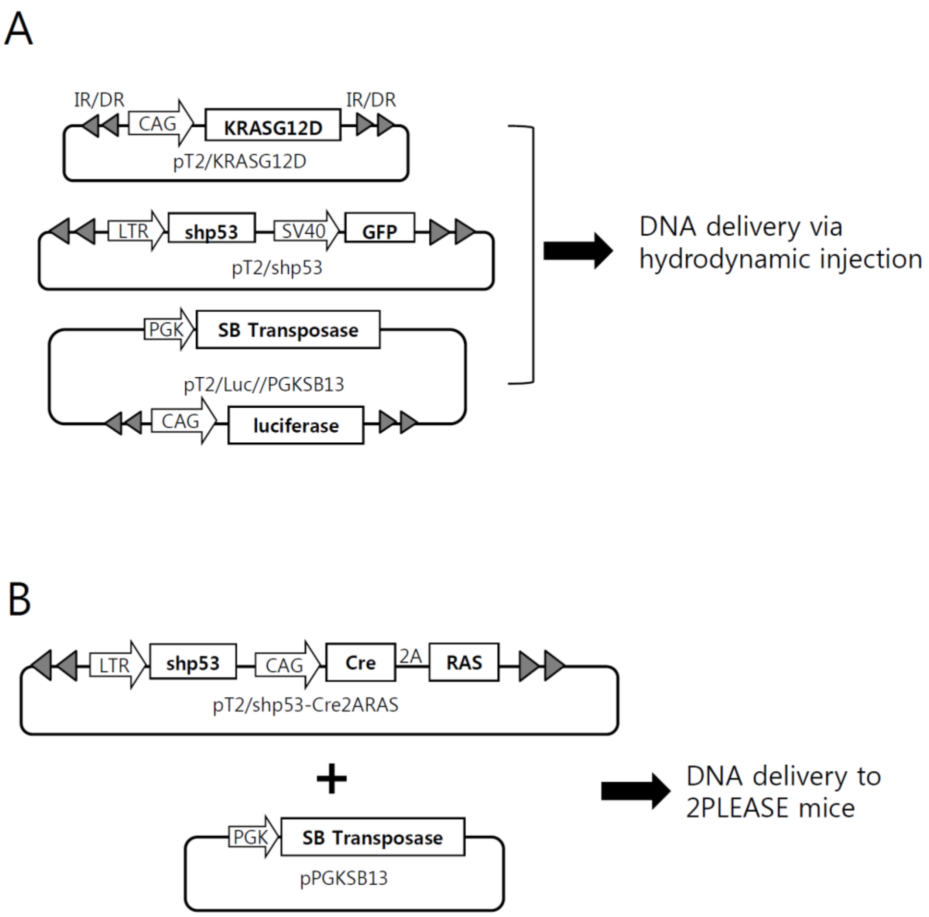

Supplementary Figure S5. Plasmids used for hydrodynamic transfection

(A) pT2/KRASG12D, pT2/shp53 and pT2/Luc//PGKSB13 were mixed and hydrodynamically delivered to wild-type mice for the experiment shown in Supplementary Fig. S6. (B) pT2/shp53-Cre2ARAS and pPGKSB13 were hydrodynamically delivered to 2PLEASE mice for the experiment shown in Fig. 5.

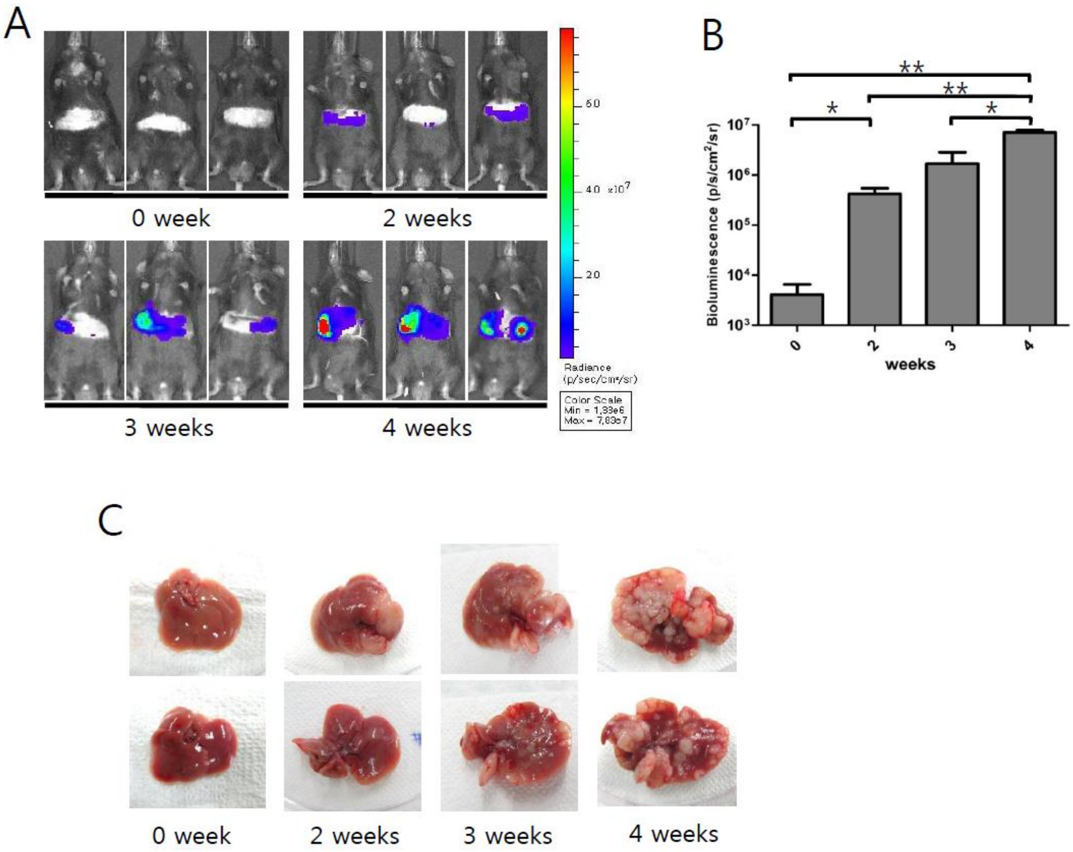

Supplementary Figure S6. Correlation between bioluminescent signal and tumor size.

(A) Following hydrodynamic delivery of pT2/KRASG12D, pT2/shp53 and pT2/Luc//PGKSB13 (see Supplementary Fig. S5), tumor growth in wild-type mice was monitored via bioluminescence imaging at 0, 2, 3 and 4 weeks. Imaging at 0 week was performed immediately before the hydrodynamic injection. (B) Graphical representation of average bioluminescent signals at the indicated time points following hydrodynamic injection. Single asterisks indicate $p < 0.05$ and double asterisks indicate $p < 0.01$. (C) Gross morphology of livers harvested at 0, 2, 3 and 4 weeks post-hydrodynamic injection.

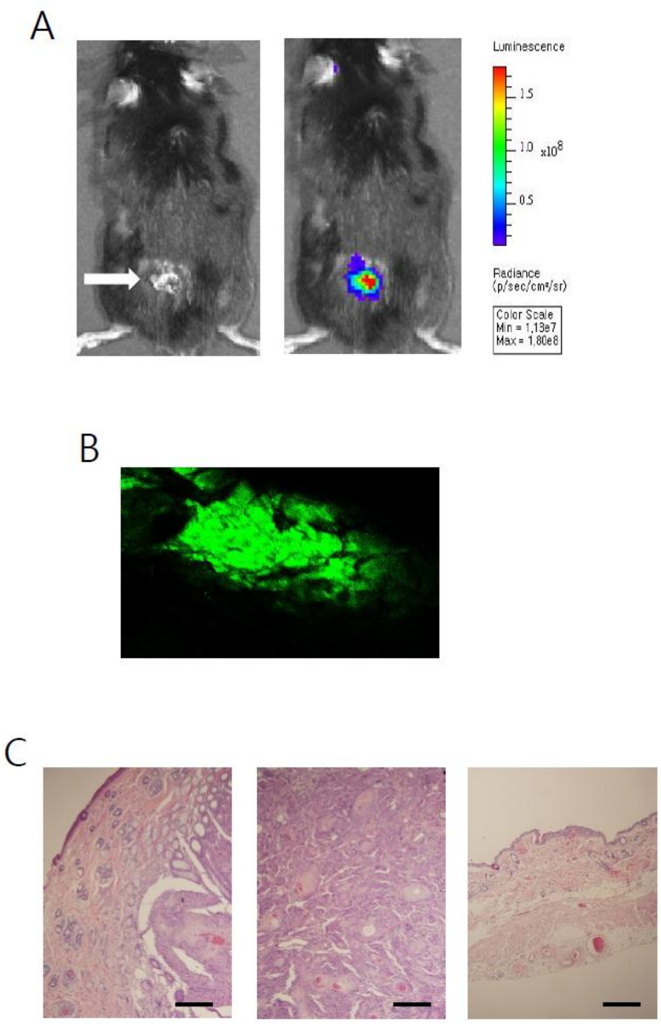

Supplementary Figure S7. A skin tumor was observed in a 2PLEASE; R26-Cre-ER^{T2} mouse.

(A) Although most double-transgenic mice showed no tumors in the skin until 8 months following topical treatment with tamoxifen, a skin tumor was found in a single mouse (left panel). The mouse happened to have a severe wound on the tamoxifen treated skin before the tumor was noticed. A strong bioluminescent signal was detected from the tumor (right panel). (B) *In vivo* fluorescence imaging revealed EGFP expression from the tumor, confirming the transgene expression. (C) H&E staining of the tumor (left and middle panels) and a normal area of skin from the same mouse (right panel). Scale bars, 100 μm.

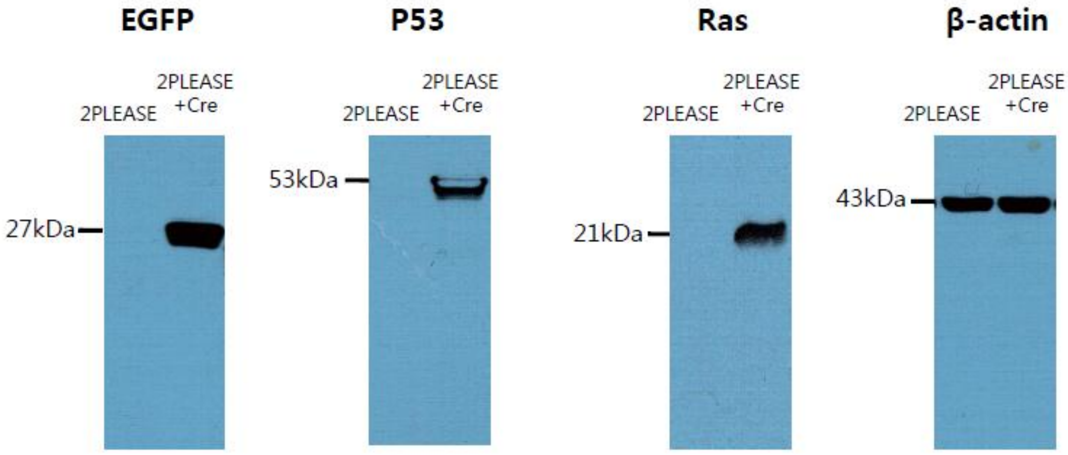

Supplementary Figure S8. Uncropped images of Figure 1C.

NIH3T3 cells were transfected with the 2PLEASE plasmids in the presence or absence of Cre. Transfected cells were harvested and Western blotting experiments were performed using anti-EGFP, anti-p53 and anti-Kras, and anti-β-actin, respectively.
